# Similarity Graph-Based Camera Tracking for Effective 3D Geometry Reconstruction with Mobile RGB-D Camera

**DOI:** 10.3390/s19224897

**Published:** 2019-11-09

**Authors:** Jaepung An, Sangbeom Lee, Sanghun Park, Insung Ihm

**Affiliations:** 1 Department of Computer Science and Engineering, Sogang University, Seoul 04107, Korea; ajp5050@sogang.ac.kr; 2 Devsisters Corp., Seoul 06019, Korea; sangbeom.lee@devsisters.com; 3 Department of Multimedia, Dongguk University, Seoul 04620, Korea; mshpark@dongguk.edu

**Keywords:** mobile RGB-D camera, 3D geometry reconstruction, similarity graph, 6-DOF pose estimation, 3D scene modeling, mixed reality

## Abstract

In this paper, we present a novel approach for reconstructing 3D geometry from a stream of images captured by a consumer-grade mobile RGB-D sensor. In contrast to previous real-time online approaches that process each incoming image in acquisition order, we show that applying a carefully selected order of (possibly a subset of) frames for pose estimation enables the performance of robust 3D reconstruction while automatically filtering out error-prone images. Our algorithm first organizes the input frames into a weighted graph called the similarity graph. A maximum spanning tree is then found in the graph, and its traversal determines the frames and their processing order. The basic algorithm is then extended by locally repairing the original spanning tree and merging disconnected tree components, if they exist, as much as possible, enhancing the result of 3D reconstruction. The capability of our method to generate a less error-prone stream from an input RGB-D stream may also be effectively combined with more sophisticated state-of-the-art techniques, which further increases their effectiveness in 3D reconstruction.

## 1. Introduction

The reconstruction of 3D worlds from 2D images has been a fundamental challenge in computer graphics and vision for decades. Its importance is equally apparent in the fields of virtual, augmented, and mixed reality where real geometry must be naturally mingled with virtual geometry. Since the Microsoft Kinect sensor became available in 2010, the direct dense methods of visual odometry and simultaneous localization and mapping (SLAM) have shown their potential for extracting 3D geometry models from RGB-D image streams captured by consumer-grade cameras (refer to the recent survey article [1] for notable results). The traditional visual odometry and SLAM approaches used for 3D reconstruction have mostly focused on real-time online computation. When a current frame is taken to estimate its camera pose, it is generally registered against the previous frame (*frame-to-frame*) or the geometry accumulated thus far in the form of signed distance fields or surfel maps (*frame-to-model*). The global relationships between captured frames may be considered through either bundle adjustment [2] or pose-graph optimization [3,4], optimizing the pose estimation errors across frames or distributing them across the graph.

In this work, we basically assume the use of a color-depth sensor with low spatial resolution and limited depth precision, such as the mobile sensor on the Google Tango-enabled smartphone, which suffers easily from sensor errors, noises, blurs, and distortions. Under this situation, if high-quality geometry reconstruction is of more concern, it is quite desirable for a user to first scan along areas of interest rather densely enough to acquire sufficient RGB-D data, and carefully select a set of (not necessarily all) image frames from the scan that would allow faithful reproduction of 3D objects. In particular, if the real-time online camera tracking is not mandatory, there is no need to process the RGB-D stream in its given order or for all frames to participate in reproducing the 3D geometry despite any possible defects in the images.

In this paper, we experiment with a different approach for 3D geometry reconstruction that is free from these restrictions. Given an input RGB-D stream, in an aim to extract the best-possible sequence of (possibly a subset of) input frames for stable camera tracking and 3D reconstruction, we build a graph, called the *similarity graph*, each of whose edges indicates the similarity of the pair of incident frames with respect to pose estimation. Then, a set of pairs of frames that enable a robust 3D reconstruction are carefully selected from the graph along with the pose estimation order by traversing a *maximum spanning tree* in the graph.

In addition to the generation of an effective set of input frames and their registration order, the presented similarity graph scheme also automatically removes from consideration those frames that may introduce intolerable errors in the results of pose estimation and 3D reconstruction. This may disconnect the similarity graph, resulting in multiple separate components. Therefore, we perform an additional process, called the *component-to-component* camera tracking, to connect them as much as possible.

To evaluate the effect of the similarity graph clearly, our method currently employs a simple frame-to-frame tracking model for the pose estimation, which can be easily modified to include advanced mechanisms, such as the frame-to-model tracking, bundle adjustment, and pose-graph optimization. This does not mean that the presented similarity graph technique is orthogonal to these sophisticated mechanisms because our method may also be easily combined with them to enhance their effectiveness by providing them with fewer error-inducing input streams.

## 2. Previous Work

The introduction of the Microsoft Kinect camera led to the possibility of using commodity RGB-D cameras for effective 3D reconstruction. In their seminal work, Newcombe et al. [5] presented a frame-to-model camera tracking system that successfully manipulated the captured depth information to reproduce 3D geometry in a volumetric signed distance field. Since then, several follow-up studies have scanned large-scale areas [6,7] and devised an efficient representation of the memory-intensive volume data structure [8,9]. Unlike the Kinect system, different pose estimation models were proposed that use photometric information [10,11] and both photometric and geometric information [12] to formulate the cost function for direct dense visual odometry. Since then, variants of these approaches were applied to build the RGB-D camera-based tracking and reconstruction systems.

In order to cope with the pose estimation errors that are inevitably accumulated significantly over time, more sophisticated global optimization mechanisms such as bundle adjustment [2] and pose-graph optimization [3,4] were applied to maintain the global consistency between camera pose estimates. The spatial relation between a selection of keyframes was estimated using the image features in the input RGB-D images (e.g., using randomized ferns [13]), which allowed a pose graph to be incrementally optimized for a consistent pose estimation [14,15]. Statistical methods, such as the surfel-based matching likelihood measure [16] and the entropy-based likelihood estimate [17], were also explored to evaluate the spatial relations between the input frames.

The geometric relationships between submaps respectively constructed from chunks of temporarily adjacent frames in the input stream were explored for more accurate large-scale reconstruction [18,19,20,21,22,23]. Several surfel-based, frame-to-model tracking methods were also proposed (e.g., [16,24]) to efficiently represent the 3D models incrementally accumulated during the camera tracking. This approach was then combined with various nonrigid dense map deformation techniques for maintaining globally consistent maps [25,26,27]. All these research efforts have culminated into the recent development of several open-source camera tracking systems (e.g., [22,27,28]).

Note that, similarly to our method that builds a maximum spanning tree from the similarity graph, some previous works also constructed a minimum spanning tree over the pose graph but in the context of global optimization or loop closures for selecting appropriate keyframes [28,29,30]. On the other hand, the maximum spanning tree is used in the presented method to reorder frames in the input sequence for stable pose estimations.

## 3. Preliminaries: 6-DOF Camera Pose Estimation

As input data, we assume an RGB-D sequence such that each frame at time *i* (i=0,1,2,…) provides a pair of aligned images $F_{i}=\left(I_{i},D_{i}\right)$, consisting of an intensity image $I_{i}\left(u\right)$ and a depth map $D_{i}\left(u\right)$, respectively seen through every pixel $u\in U\subseteq R^{2}$. For a set of valid pixels $u_{m}$ ($m=1,2,\ldots ,n_{p})$ of the *i*th frame that are used in the 6-DOF camera pose estimation, let $p_{m}\in R^{3}$ ($m=1,2,\ldots ,n_{p}$) be the nearest 3D point in the *i*th frame’s camera space that is visible through $u_{m}$.

Given the 3×3 intrinsic matrix *K* of the depth sensor, each point can be described as $p_{m}=D_{i}\left(u_{m}\right)K^{-1}{\left(u_{m}^{⊤}\;\;1\right)}^{⊤}$. Reversely, the perspective projection from an arbitrary 3D point p in the camera space to a pixel u becomes u=μ(p)=π(Kp), where $\pi \left(x,y,w\right)={\left(\frac{x}{w},\frac{y}{w}\right)}^{⊤}$ is the dehomogenization operator in $R^{2}$. Let $\xi ={\left(\omega^{⊤}\;\upsilon^{⊤}\right)}^{⊤}$ denote the 6-vector of coordinates in the Lie algebra se(3) with $\omega \in R^{3}$ and $\upsilon \in R^{3}$ respectively determining the rotation and the translation. Then, the motion parameter ξ generates a rigid transformation matrix T(ξ)∈SE(3) through the exponential map $T\left(\xi \right)=exp\left(\hat{\xi }\right)$, where, for the skew-symmetric matrix operator ${\left[\cdot \right]}_{\times }$, $\hat{\xi }=\left[\begin{array}{cc} {\left[\omega \right]}_{\times } & \upsilon \\ 0^{⊤} & 0 \end{array}\right].$

The goal of the *frame-to-frame* pose estimation is to estimate the rigid transformation $T_{ij}\in \mathrm{SE}\left(3\right)$ from the camera space of the *i*th frame to that of the *j*th frame based upon $F_{i}$ and $F_{j}$. This is done by, from an initial guess $T_{ij}^{\left(0\right)}$, iteratively improving the current estimate $T_{ij}^{\left(k\right)}$ until convergence such that $T_{ij}^{\left(k+1\right)}\leftarrow T\left(\xi_{min}\right)T_{ij}^{\left(k\right)}$, where $\xi_{min}$ is obtained by solving a minimization problem that is defined shortly. Consider the image warping function that maps the *m*th pixel $u_{m}$ of the *i*th frame to a pixel $v_{m}$ of the *j*th frame via a given rigid-body motion $T\left(\xi \right)T_{ij}^{\left(k\right)}$. Then, it can be expressed as $v_{m}=\left(\mu \circ h_{m}\right)\left(\xi \right)$, where $h_{m}\left(\xi \right)=T\left(\xi \right)T_{ij}^{\left(k\right)}{\left(p_{m}^{⊤}\;1\right)}^{⊤}$. (Note that the warping process, defined with respect to each pixel $u_{m}$, is a function of ξ.) If we define the error vector function $g\left(\xi \right)={\left(\;\;\;\;\ldots \;\;\;\;g_{m}\left(\xi \right)\;\;\;\;\ldots \;\;\;\;g_{m+n_{p}}\left(\xi \right)\;\;\;\;\ldots \;\;\;\;\right)}^{⊤}\in R^{2n_{p}}$, where

(1) $$g_{m}\left(\xi \right)=\sqrt{w_{I}\left(u_{m}\right)}\left\{\;\left(I_{j}\circ \mu \circ h_{m}\right)\left(\xi \right)-\left(I_{i}\circ \mu \right)\left(p_{m}\right)\;\right\}$$

and

(2) $$g_{m+n_{p}}\left(\xi \right)=\lambda \sqrt{w_{D}\left(u_{m}\right)}\left\{\;\left(D_{j}\circ \mu \circ h_{m}\right)\left(\xi \right)-{\left[h_{m}\left(\xi \right)\right]}_{z}\;\right\}$$

for $m=1,2,\ldots ,n_{p}$, then the new estimate $T_{ij}^{\left(k+1\right)}$ can be obtained by optimizing the cost function $g{\left(\xi \right)}^{⊤}g\left(\xi \right)$:

(3) $$\xi_{min}=\arg\min_{\xi }\;g{\left(\xi \right)}^{⊤}g\left(\xi \right).$$

In this objective function, $w_{I}\left(\cdot \right)$ and $w_{D}\left(\cdot \right)$ are the respective weights obtained to reduce the negative effect of outliers, λ is the relative gain for the depth component, and the operator ${\left[\cdot \right]}_{z}$ returns the *z* coordinate of a 3D point. While any feasible method (such as the one presented in, for instance, [5,10,11]) may be applied to implement the motion estimation process, we use a slight variant of the method by Tykkälä et al. [12], in which the weights in the iteratively re-weighted least squares formulation are computed using the *t*-distribution as proposed by Kerl et al. [17].

## 4. Similarity Graph-Based Camera Pose Estimation

The proposed algorithm proceeds in four steps: (i) estimation of similarity measure between frames, (ii) construction of similarity graph, (iii) extraction of maximum spanning tree, and (iv) pose estimation via tree traversal. In this section, we first explain each step in detail, and show how this basic algorithm can be extended to improve its performance further in the next section.

### 4.1. Similarity Measure between Two Frames

Given two images $F_{i}$ and $F_{j}$ captured at two arbitrary times *i* and *j*, we are to estimate the relative rigid-body motion $T_{ij}$, which maps the camera space of the *i*th frame (source frame) to that of the *j*th frame (target frame), by optimizing the cost function described in Section 3. As is well known, the stability and accuracy of the solution process are greatly influenced by how close the initial guess $T_{ij}^{\left(0\right)}$ is to the solution $T_{ij}$. In general, the identity matrix I∈SE(3) is selected as an initial value because there is no obvious better alternative. When a frame-to-frame tracking method is applied between two *temporarily consecutive* frames, however, such a choice may lead to a poor estimation when the camera has moved fast at a high angular velocity between the frames, resulting in abrupt view changes and annoying motion blurs in the captured images. This is mainly because, in such case, the unknown rigid-body motion $T_{ij}$ is located far from the null motion *I* in the search space SE(3).

If we are free to choose the pairs of frames to be registered to each other, it is very advisable to select the pairs whose images are as *similar* as possible. For such pairs, whose relative rigid transformation would be close to the initial guess (which is set to the null motion), we observe that even the simple, drift-prone, frame-to-frame tracking tends to produce quite satisfactory pose estimations. Under this observation, we define *the similarity between the two frames $F_{i}$ and $F_{j}$ over a rigid-body motion T* as the ratio between the cardinalities of two pixel sets $P_{valid}\left(F_{i}\right)$ and $P_{matched}\left(F_{i},F_{j},T\right)$:

(4) $$\sigma \left(F_{i},F_{j},T\right)=\frac{|\;P_{matched}\left(F_{i},F_{j},T\right)\;|}{|\;P_{valid}\left(F_{i}\right)\;|},$$

where $\sigma (F_{i},F_{j},T)$ ranges from 0 to 1 as explained immediately below. First, $P_{valid}\left(F_{i}\right)$ indicates the set of pixels of $F_{i}$ that are found *valid* and thus may participate in the process of camera pose estimation. Note that problematic pixels often appear in various forms in the captured RGB-D images. Above all, depth sensors in general generate pixels of invalid values for out-of-range readings, which should be eliminated from consideration. In addition, we get rid of pixels further that may cause numerical instability during the pose estimation computation. In particular, to remove those pixels with possibly faulty depth values, the normal and contour filtering techniques, proposed by An et al. [31], are applied after the usual bilateral filtering.

Second, $P_{matched}\left(F_{i},F_{j},T\right)$, which is a subset of $P_{valid}\left(F_{i}\right)$, represents the set of valid pixels of $F_{i}$ that after *warped* from $F_{i}$ to $F_{j}$ through *T*, have both similar intensity and depth values in $F_{j}$. Ideally, the pixel values of $u\in P_{valid}\left(F_{i}\right)$ must be the same as those of the corresponding pixel $v_{u}$ of $F_{j}$ if $T=T_{ij}$, where $v_{u}=\mu \left(T{\left(p_{u}^{⊤}\;1\right)}^{⊤}\right)$ and $p_{u}=D_{i}\left(u\right)K^{-1}{\left(u^{⊤}\;\;1\right)}^{⊤}$. In reality, however, since the captured RGB-D images often contain noisy and faulty pixel values, the rigid transformation *T* is only an approximate for $T_{ij}$, and the 3D objects in the scene are not ideally diffusive, they usually do not coincide exactly. If *T* is a rather good estimate, however, there is a good chance that there exists a pixel in the neighborhood of $v_{u}$ in the pixel space of $F_{j}$ whose intensity and depth values are very similar to those of u.

To reflect this uncertainty, we regard that a pixel u in $F_{i}$*matches* some pixel in $F_{j}$ with respect to *T* and an image kernel of fixed odd size $n_{ker}$, that is, $u\in P_{matched}\left(F_{i},F_{j},T\right)$, if, for $v_{u}=v\left(x,y\right)$, there exists at least one neighboring pixel v(x+k,y+l) in $F_{j}$ with $-\frac{n_{ker}-1}{2}\leq k,l\leq \frac{n_{ker}-1}{2}$ such that

- $v\left(x+k,y+l\right)\in P_{valid}\left(F_{j}\right)$, i.e., v(x+k,y+l) is valid in $F_{j}$,
- $|\;I_{j}\left(v\left(x+k,y+l\right)\right)-I_{i}\left(u\right)\;|<\tau_{I}$ for some threshold $\tau_{I}$, and
- $|\;D_{j}\left(v\left(x+k,y+l\right)\right)-{\left[T{\left(p_{u}^{⊤}\;\;1\right)}^{⊤}\right]}_{z}\;|<\tau_{D}$ for some threshold $\tau_{D}$.

**Figure 1:**
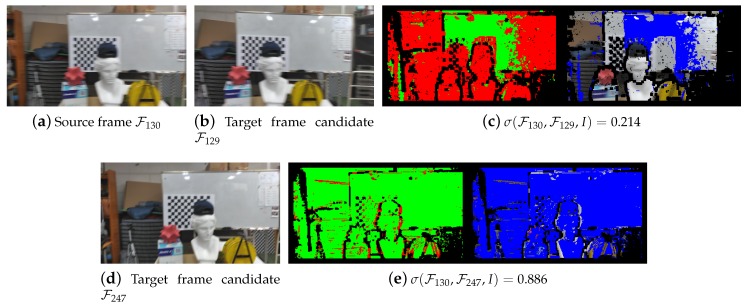
Selection of a target frame for a given source frame. While capturing a stream of RGB-D images of 320×180 pixels using a Google Tango-enabled smartphone, there happened to be a jerky motion between the 129th and 130th frames, which was confirmed by the low similarity measure (**c**). The presented method found that the 247th frame is in fact the best candidate as a target frame, for which the similarity measure increased markedly (**e**). By registering the source frame $F_{130}$ against the upcoming frame $F_{247}$, not the preceding frame $F_{129}$, we could actually avoid a significant pose estimation error. In (**c**) and (**e**), the green and red colors indicate the valid pixels of the source frame $F_{130}$ that respectively matched and did not match some pixels of the corresponding target frame candidates. On the other hand, the blue color in the target frame candidates represents the pixels that were matched by some source frame pixels. In our experiments, we set the similarity measure parameters as follows: $n_{ker}=5$, $\tau_{I}=10/255$, and $\tau_{D}=4$ (mm).

It is known that the convergence of the Gauss–Newton method, which is used to implement our optimizer, can suffer when the optimal objective value is relatively large [32]. Note the resemblance between the residual errors in the cost function of Section 3 and the similarity criteria. When a pair of frames with high similarity are chosen, the iteration will tend to start with a relatively small objective value $g{\left(\xi_{0}\right)}^{⊤}g\left(\xi_{0}\right)$, ensuring numerically more stable optimization. See Figure 1 for some examples of the similarity measure.

### 4.2. Construction of Similarity Graph

Now, for a given stream of $n_{fr}$ frames $F_{i}=\left(I_{i},D_{i}\right)$$\left(i=0,1,\ldots ,n_{fr}-1\right)$, we build a weighted undirected *similarity graph*G=(V(G),E(G)), where V(G) is the set of vertices respectively representing the $n_{fr}$ frames, E(G) is the set of edges connecting each pair of frames, and the weight of an edge $\left(F_{i},F_{j}\right)\;\left(i>j\right)$ is the similarity value $\sigma (F_{i},F_{j},T)$ for a given rigid transformation *T*. Two things need to be noted about the similarity graph. First, strictly speaking, the similarity measure on a pair of frames is not commutative in general, i.e., $\sigma \left(F_{i},F_{j},T\right)\neq \sigma \left(F_{j},F_{i},T^{-1}\right)$, implying the graph must be directed. However, in order to reduce the computational burden of constructing the similarity graph, we confine ourselves to registering frames to the temporarily precedent frames only, which still produces sufficiently good results.

Second, whereas evaluating the weight function requires the matrix *T*, which must ideally be $T_{ij}$, there is no information at all on the rigid-body motion in particular when the image sequence has just been inputted. When the similarity graph is constructed for the first time, we use the null transformation *I* as *T*. In this case, the pairs of frames whose corresponding cameras have closer positions and orientations in the global space tend to have higher similarity measures. Therefore, when edges with larger weights are selected for pose estimation, as will be explained shortly, they usually produce less error-prone estimates for the wanted rigid transformations.

### 4.3. Extraction of Maximum Spanning Tree

Recall that when the camera pose is tracked along the usual, temporarily linear trajectory, a single substantial error involved in registering a specific pair of consequent frames may cause severe drifts for all subsequent frames (frame-to-frame tracking) or accumulate incorrect geometry information into the model being built (frame-to-model tracking). Under the intuition that selecting higher weighted edges will result in more reliable registrations for the corresponding frame pairs, we are naturally led to the greedy strategy in which the edges are selected from the similarity graph on the order of non-increasing weights until all vertices (frames) are included without forming a cycle. Such a choice can be made by constructing a maximum spanning tree in the graph, which is done by negating the edge weights and finding a minimum spanning tree. In this work, we implemented the priority-queue-based version of Kruskal’s algorithm that finds a desirable tree in O(|E(G)|+αlog|V(G)|) time, where α is the number of graph edges not longer than the longest edge in the minimum spanning tree [33].

**Figure 2:**
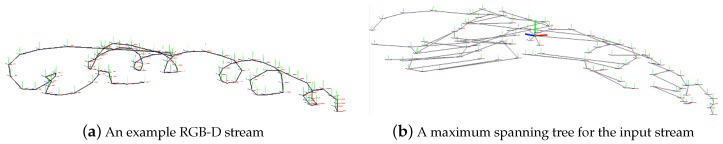
Construction of a maximum spanning tree from an input RGB-D stream. From the similarity graph that was built for the stream shown in (**a**), our method found a maximum spanning tree whose root frame is marked in thicker lines in (**b**). Note the difference in the general appearance of the camera trajectories estimated through the linear path and the maximum spanning tree, respectively.

After a maximum spanning tree is obtained, we select the *root frame* whose camera space becomes the global space common to all frames. It is highly probable that the longer the distance from a frame to the root frame is, the more numerical errors are accumulated while transforming the frame’s camera space to the global space. Therefore, we find a *center* $F_{c}$ of the tree whose greatest distance to other frames in the maximum spanning tree is minimal: $c=\arg\min_{i}\max_{j\neq i}d_{T_{G}}\left(F_{i},F_{j}\right)$, where $d_{T_{G}}\left(\cdot ,\cdot \right)$ is the distance (i.e., the number of edges) between two frames in the tree *T*. Refer to Figure 2 to see an example of maximum spanning tree that was built from an RGB-D sequence.

### 4.4. Pose Estimation through Tree Traversal

Finally, we traverse the maximum spanning tree in depth-first search manner, performing the frame-to-frame pose estimation for each visited edge which represents a pair of source-target frames. During the tree traversal, the camera space of each frame is converted to the global space through the transformation obtained by accumulating the relative camera motions from the frame to the root. Here, since the pairs of frames for which the camera poses are sought are known a priori, the camera tracking computation can easily be parallelized.

**Figure 3:**
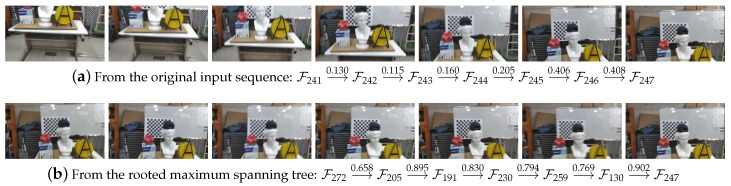
Extraction of a frame sequence that is more effective for camera tracking. Compare the two frame sequences that respectively reach the same frame $F_{247}$. While the frames were captured, the camera was moved rather fast between the 241th and 247th frames as shown in (**a**), for which a naïve application of the frame-to-frame tracking technique was destined to fail. In contrast, the similarity graph method was able to suggest a frame sequence, displayed in (**b**) that enabled the same frame-to-frame pose estimation method to track the camera more accurately. In each figure, the similarity measures between the respective frame pairs are shown.

Note that the edges with low similarity measures may introduce intolerable pose estimation errors although they may happen to be selected during the tree construction, depending on how the input stream was captured. In that case, we often witness poor pose estimations between the corresponding pairs of frames, resulting in visually annoying artifacts in the resulting 3D reconstruction. Therefore, when the tree is built, our method allows for limiting the edges in the similarity graph to have edge weights greater than a minimum threshold value $\tau_{good}$ (e.g., $\tau_{good}=0.8$). Depending on the input stream, this restriction may disconnect the similarity graph, producing a forest of trees, each of which is itself a maximum spanning tree. We discuss how to handle the multiple connected components of the similarity graph in Section 5.2. See Figure 3 for a branch of an example tree generated by our method.

## 5. Extending the Idea of a Similarity Graph

### 5.1. Local Repair of a Maximum Spanning Tree

Despite the selection of frame pairs having as high similarity as possible during the construction of the maximum spanning tree, it is often that the rigid transformations estimated for some edges are not sufficiently accurate. One reason for this finding is that some edges connecting irrelevant frames were wrongly chosen because of the inaccurate approximations of the similarity measure. Recall that, when the similarity is evaluated for a given pair of frames, the identity matrix is initially used as the rigid transformation between them because there is no better choice. However, this may cause a serious problem when there was a nontrivial translational and/or rotational motion between the two frames, reducing the reliability of the similarity measure. On the other hand, the rigid transformation estimated for each tree edge during the tree traversal is usually a better approximation for the wanted transformation than the identity matrix.

**Figure 4:**
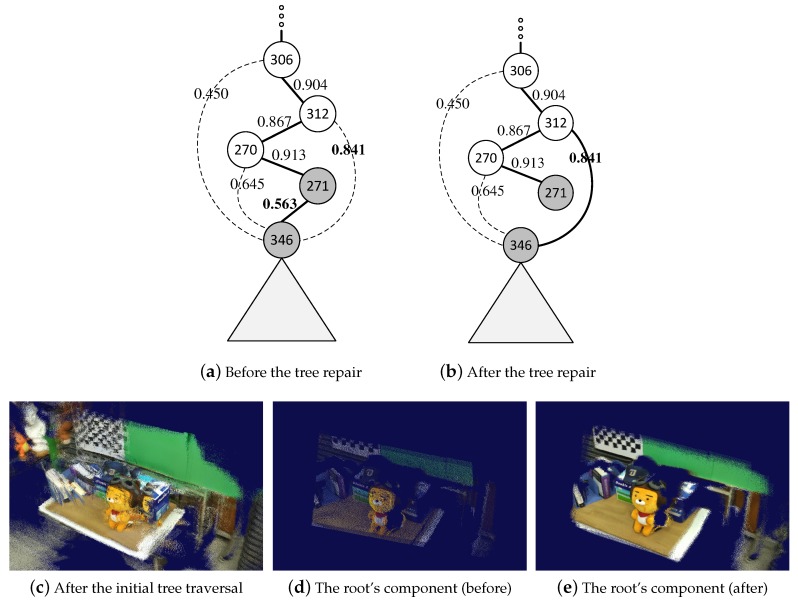
Repair of the maximum spanning tree. The rigid transformations estimated using the initial maximum spanning tree can increase the reliability of the similarity evaluation, which in turn allows for selecting *more similar* pairs of frames in the tree repair process. (**a**) and (**b**) illustrate the situation where the currently visited edge $\left(F_{346},F_{271}\right)$ is replaced by a new one $\left(F_{346},F_{312}\right)$. Then, (**c**) displays the point cloud initially produced with respect to the entire 662 frames of an input RGB-D stream. The visually annoying artifact was mainly due to the inaccurate approximations of the similarity measure, causing some irrelevant pairs of frames to be selected. When those edges with similarity measures greater than $\tau_{good}=0.75$ were used for 3D reconstruction, only two frames were left in the connected component containing the root frame (**d**). This was because most edges near the root node happened to have a low similarity measure. When the tree was repaired with the repair parameter $\tau_{repair}=0.8$, a more robust 3D reconstruction was possible where the size of the connected component grew to 56 frames (**e**).

In our method, we (optionally) traverse the tree again and repair it, if possible, using the more reliable estimates of the rigid transformations. Given a maximum spanning tree, let $F_{i}$ and $F_{j}$ be the child (source) and parent (target) frames, respectively, of an edge currently being visited. To estimate the appropriateness of using this pair of frames in the pose estimation, we evaluate the similarity measure $\sigma (F_{i},F_{j},{\hat{T}}_{ij})$ again using the rigid transformation ${\hat{T}}_{ij}$ estimated in the first round. If the new similarity measure is less than a given threshold $\tau_{repair}$, we examine the proper ancestors $F_{k}$ of $F_{j}$ up to, say, three levels, evaluating the respective similarity measures $\sigma (F_{i},F_{k},{\hat{T}}_{ik})$, where ${\hat{T}}_{ik}$ can be approximated by accumulating the rigid transformations along the path from $F_{i}$ to $F_{k}$ (refer to (a) and (b) of Figure 4).

If the largest one among the reevaluated measures is greater than $\tau_{repair}$, we delete the edge between $F_{i}$ and $F_{j}$ from the maximum spanning tree, and instead insert a new one between $F_{i}$ and the corresponding ancestor $F_{k^{*}}$ (e.g., $F_{312}$ in Figure 4b). When the rigid transformation $T_{ik^{*}}$ is to be estimated for the new edge, we use the rigid transformation accumulated from $F_{i}$ to $F_{k^{*}}$ as an initial value for the iterative optimization process, which usually results in a more accurate pose estimate than using the identity transformation. Once the repair process is done, we use only those edges with similarity measures greater than $\tau_{good}$ for 3D reconstruction, as explained in Section 4.4. See Figure 4 for an example of the tree repair.

### 5.2. Component-Wise Camera Tracking

When the parameter $\tau_{good}$ is set to a high value, say, 0.85, and the edge set of the similarity graph is restricted to those with weights above $\tau_{good}$, the graph tends to get disconnected into multiple connected components. In such case, the quality of the point cloud generated for each component is quite high, and thus it is desirable to align the major components to each other to form a larger one.

**Figure 5:**
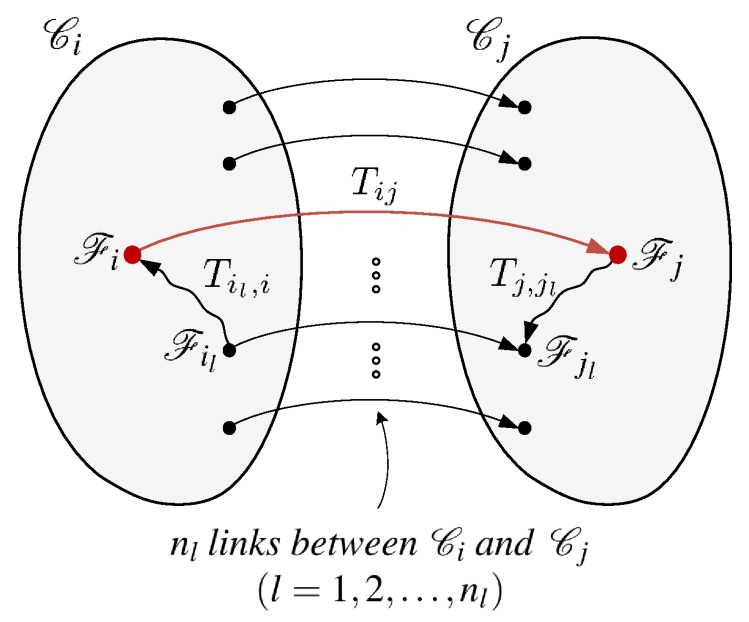
Component-to-component camera tracking. Given two connected components $C_{i}$ and $C_{j}$ whose root frames in the respective maximum spanning trees are $F_{i}$ and $F_{j}$, the rigid-body motion $T_{ij}$ that will align the two components in the common space is estimated by collectively using the next best available frame pairs $\left(F_{i_{l}},F_{j_{l}}\right)$ connecting them. Note that the two rigid transformations $T_{i_{l},i}$ and $T_{j,j_{l}}$ can be derived from the respective trees.

Let $C_{i}$ and $C_{j}$ be two connected components of a similarity graph, for each of which the 3D geometry has successfully been reproduced using the similarity graph method (see Figure 5). Assume further that $F_{i}$ and $F_{j}$ are the root frames of the minimum spanning trees that were built in the respective components. Being separated as two components means that there was not a single highly reliable edge between them that allows for deriving the rigid transformation $T_{ij}$ between the two frames. However, there often exist edges that connect the two components with a fair, although not sufficient, amount of similarity. Consider those edges whose similarity measures are greater than a given threshold $\tau_{fair}$, which is set to a value smaller than $\tau_{good}$ (e.g., $\tau_{fair}=0.7$). Then, although each of them may not lead to a sufficiently accurate *frame-to-frame* camera tracking, they often, although not always, can be collectively used to estimate the relative rigid-body motion $T_{ij}$.

In this *component-to-component* tracking approach, for each such edge $\left(F_{i_{l}},F_{j_{l}}\right)$ ($l=1,2,\ldots ,n_{l}$), every valid pixel $u_{m}$ of the $i_{l}$th frame contributes to the error vector g(ξ) in basically the same way as the frame-to-frame tracking discussed in Section 3. Again, let $p_{m}$ be the point in the $i_{l}$th frame’s camera space that corresponds to $u_{m}$. Then, the only difference in the formulation of the error function for finding $T_{ij}$ is the way of transforming $p_{m}$ to the $j_{l}$th frame’s camera space, where the warping function now becomes $v_{m}=\left(\mu \circ h_{m}^{l}\right)\left(\xi \right)$ where $h_{m}^{l}\left(\xi \right)=T_{j,j_{l}}T\left(\xi \right)T_{ij}^{\left(k\right)}T_{i_{l},i}{\left(p_{m}^{⊤}\;1\right)}^{⊤}$. It should be noted that, during the minimization of the new cost function, the Jacobian matrix $J_{g}\left(\xi \right)\in R^{2n_{tp}\times 6}$, where $n_{tp}$ is the total number of pixels collected from the $n_{l}$ frames in $C_{i}$, must be constructed slightly differently using the modified 3×6 Jacobian of $h_{m}^{l}\left(\xi \right)$, which can be expressed as

(5) $$\begin{array}{ccc} J_{h_{m}^{l}}\left(\xi \right) & = & R\left(T_{j,j_{l}}\right)\left[\begin{array}{cc} I_{3\times 3} & -{\left[{\bar{p}}_{m}\right]}_{\times } \end{array}\right]. \end{array}$$

**Figure 6:**
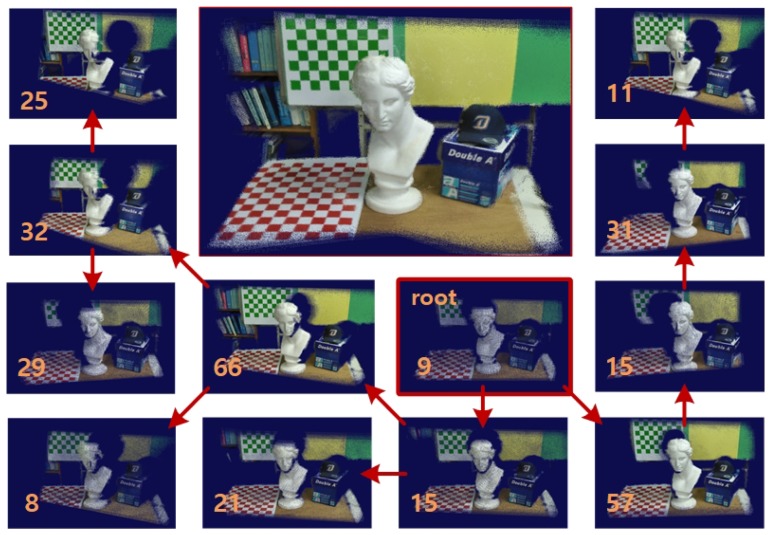
Merge of separate components. When the similarity graph method was applied to an input stream of 843 frames with $\tau_{good}=0.75$, we obtained 12 separate components. To combine them as much as possible, we first repaired each component using $\tau_{repair}=0.8$, and built a component graph whose edges are those with the highest similarity measure between the components. Then, after finding a maximum spanning tree each of whose vertices are shown in the small figure with its frame number, we performed the component-wise camera tracking with $\tau_{fair}=0.6$ while traversing the tree, computing the rigid transformations between the components. As a result, we could collect all components into a common space as shown in the large figure. Note that only 319 frames out of 843 were actually used to reproduce the final geometry.

Here, $R(T_{j,j_{l}})$ is the 3×3 rotation matrix of $T_{j,j_{l}}$, $I_{3\times 3}$ is the 3×3 identity matrix, and ${\bar{p}}_{m}\in R^{3}$ is the point mapped from $p_{m}$ via $T_{ij}^{\left(k\right)}T_{i_{l},i}$. See Figure 6 for an example of the component-wise camera tracking.

## 6. Experiments

To demonstrate its effectiveness and applicability, the presented similarity graph scheme was tested with several RGB-D sequences, where all the test datasets, including those shown in the previous sections, were produced by storing into files the live RGB-D streams of 320×180 pixels, captured using a Lenovo Phab 2 Pro smartphone. Compared to the Microsoft Kinect v2 sensor, the used mobile RGB-D sensor tended to suffer more from the limited depth precision and low pixel resolution. In addition, we often observed visually annoying temporal/spatial mismatches between the pixels of the intensity and depth images presumably due to the difference in their image acquisition rates, making the 3D reconstruction process more challenging. As mentioned in the Introduction section, for high-quality 3D reconstruction, our method is oriented to choose best possible frames and their camera tracking order from input sequences. Therefore, it is better suited for the input streams in which areas of interest are scanned rather densely so as to provide sufficient RGB-D information. Therefore, we found that such standard benchmark datasets as the ICL-NUIM or TUM datasets [34,35] are not best suited to evaluate our method.

### 6.1. Computational Costs

The effectiveness of the presented method only comes with the computational cost of finding the sequence of frame pairs that enables the robust and accurate camera tracking. Table 1 reveals the computational complexity of our method, where the timings were collected on a PC with an Intel Core i7-8700K CPU with 64 GB of main memory and an Nvidia GeForce GTX 1080 Ti GPU with 11 GB of graphics memory. To build a similarity graph for an input RGB-D stream of $n_{fr}$ frames, the similarity measure should be evaluated for each of the $\frac{n_{fr}\left(n_{fr}-1\right)}{2}$ pairs of frames, whose number increases quadratically with respect to the input size. In fact, this evaluation process has a high degree of parallelism, both frame-pair-wise and pixel-wise that allows efficient parallel processing. Despite our GPU implementation using the OpenCL, however, most computation time was spent in constructing the similarity graph as clearly shown in the table. Note that, for the stream of $n_{fr}$ frames, the frame-to-frame pose estimation needs to be carried out $n_{fr}-1$ times whether the similarity graph scheme is applied or not. Therefore, the naïve, drift-prone, frame-to-frame tracking can be regarded as consuming roughly the same per-frame pose estimation time as that given in the parentheses of the fifth column. Considering the substantially improved stability and accuracy achieved by our method, we believe that the increase in the amortized per-frame camera tracking cost was quite acceptable. In the final section, we discuss how the burdensome computation of the similarity graph construction can be done progressively during image capture in the future work.

### 6.2. Comparison to a Frame-to-Frame Tracking Method

**Figure 7:**
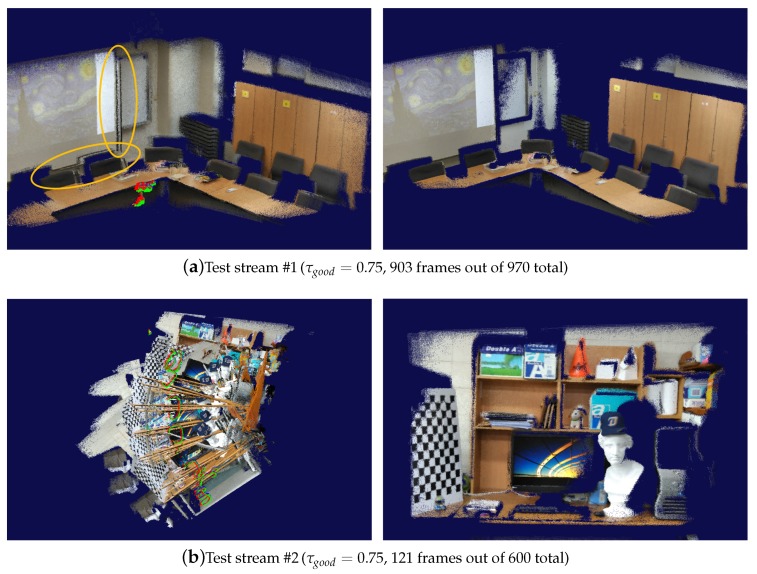
Comparison to an improved frame-to-frame tracking method. Each pair of images compare the point clouds created by the An et al.’s method [31] (**left**) and our method (**right**), respectively. As marked in ellipses in (**a**), the previous frame-to-frame technique often suffered from drifts around planar surfaces even at a normal camera speed, whereas the presented one could automatically remove the troublesome frames from consideration for camera tracking. When the camera movement was beyond its capability of adaptive error correction, as shown in (**b**), the An et al.’s method caused significant drifts of the camera poses. In contrast, when the pairs of source and target frames were selected carefully as proposed by the presented method, the simple frame-to-frame camera tracking produced quite robust results. Limiting the use of frames via the parameter $\tau_{good}$ had a nice effect of automatically filtering out those frames that may produce intolerable errors in the reconstructed 3D geometry. The frame number in the respective caption indicates the size of the largest connected component of the similarity graph, which is displayed here.

In order to see how effectively the careful selection of source-target frame pairs from an input stream improves the quality of pose estimation, we first compared our method to an extended frame-to-frame tracking method of An et al. [31], which uses a multi-level pose-error correction scheme. The experiments indicated that both methods worked well when the camera was moved smoothly and rather slowly during image capture. When the camera movement exceeded an acceptable level, however, moving irregularly in jerky motions, the An et al.’s method could no more handle such adverse situations despite its effort to correct the pose estimation errors. On the other hand, the similarity graph enabled us to find an effective set of source-target frame pairs, from which quite acceptable frame-to-frame camera tracking results were obtained without needing to use such sophisticated tools as the frame-to-model tracking and/or global pose optimization (see Figure 7).

### 6.3. Comparison to the ElasticFusion Method

We next compared our method to a state-of-the-art method, called the ElasticFusion. As proposed by Whelan et al. [27], the camera tracking system employs a surfel map-based frame-to-model tracking approach, augmented with several mechanisms like geometry deformation and local/global loop closure. Unlike ours, it was developed as a real-time online system that performs all calculations on the fly without any preprocessing. However, we carried out this analysis to understand how the effort of selecting appropriate pairs of frames from the entire RGB-D stream compares to that of applying various mechanisms to ensure the accuracy of the estimated poses while processing the input frames in acquisition order. In doing the experiment, we used the codes courteously provided by [27].

**Figure 8:**
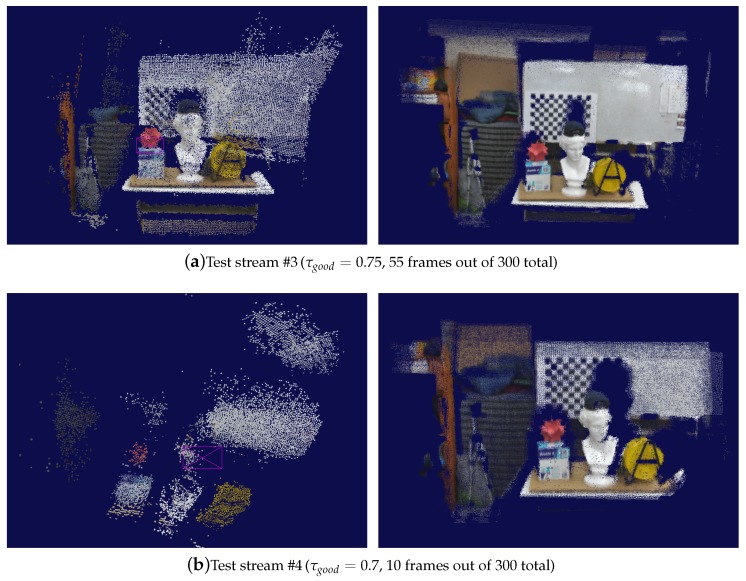
Comparison to the ElasticFusion method [27]. To see how negatively the presence of very abrupt and jerky camera motions in the RGB-D stream affects the pose estimation, we also compared our method to the performance-proven ElasticFusion system. In the usual situation, the ElasticFusion system, adopting a frame-to-model tracking, successfully reconstructed the scene. When we sporadically moved the camera very irregularly so that some camera views went outside the space of accumulated models, the ElasticFusion system (left) failed to correctly estimate the camera poses for some frames despite its several mechanisms for robust camera tracking (**a**). The situation got more aggravated as more radical camera movements were involved (**b**). In contrast, our method (right) could find the appropriate sets of source and target frames, still allowing acceptable 3D reconstructions.

As expected, under normal circumstances in which the camera movement allowed each input frame to share sufficient geometry with the accumulated model, the ElasticFusion system always completed its mission successfully. However, when we sporadically moved the camera sharply or suddenly during image capture so that the camera for some input frames looked at the space that hardly contained the accumulated model, it sometimes produced inaccurate pose estimates (see Figure 8). On the other hand, our method could still find sufficient numbers of frame pairs for stable 3D reconstruction, producing point clouds with fewer geometric errors. Note that since our method used only those frame pairs whose corresponding edges had sufficient similarity, a sparser point cloud was usually produced for the input stream containing more radical camera movements.

When the real world is scanned for 3D reconstruction, it might be necessary to generate additional RGB-D streams to supplement the poorly scanned regions. The property of being independent of the order of incoming frames implies that our method can also be used effectively to combine multiple independently scanned streams if they contain frames to each other that have enough similarity. Note that the pairs of source and target frames resulting from the similarity graph are aimed to reduce the possibility of improper pose estimations. Therefore, if a robust 3D scanning is the primary concern, the presented similarity graph scheme may also be used as a preprocessor that feeds refined RGB-D streams into the other state-of-the-art techniques like the ElasticFusion.

### 6.4. Comparison to the BundleFusion Method

We also performed a comparison with another state-of-the-art method by Dai et al. [22], called the BundleFusion that, based on the bundle adjustment framework, estimates globally optimized camera poses and produces 3D polygonal models in real-time. Our experiment showed that this global optimization framework generally performs very well in terms of both speed and scan quality. Nevertheless, for some input streams, it was desirable to filter out frames more aggressively that may introduce pose estimation errors.

**Figure 9:**
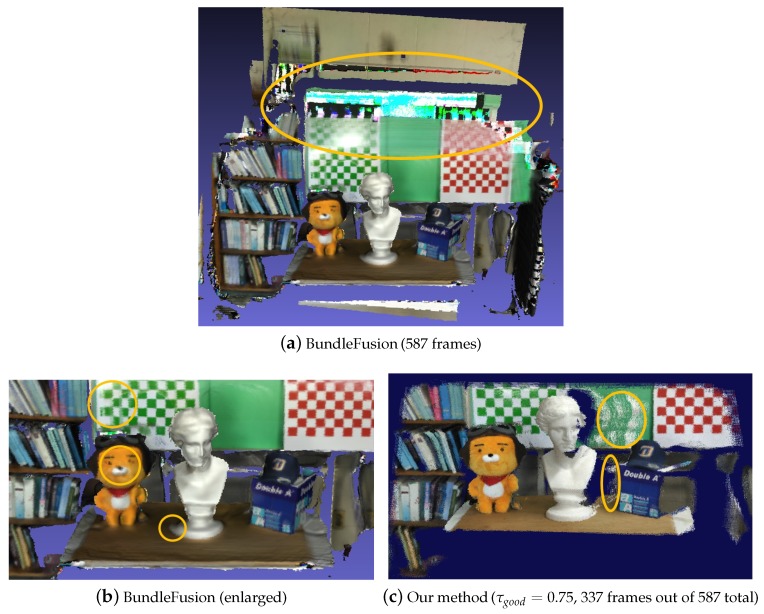
Comparison to the BundleFusion method [22]. For the test dataset of 587 RGB-D images that contained very fast and abrupt camera motion, the BundleFusion method scanned the scene robustly as intended, ensuring global model consistency. As marked in ellipse in (**a**), however, it was not easy to produce precise pose alignment against some regions corresponding to intractable camera motion mainly due to insufficient information. We also observed that trying to handle such camera motions sometimes affected local consistency negatively as marked in circle in (**b**). Rather than including as many frames as possible, our method takes a different approach where only those frames that, together, would lead to stabler 3D geometry reconstruction are selected for pose estimation; (**c**) shows the result from the similarity graph technique where 337 interrelated frames, merged from eight separate connected components of the similarity graph, were used. Note that the aliases in the point cloud marked in ellipse in (**c**) were due to the intensity/depth pixel mismatches and noises often incurred by the low-end mobile sensor, which could be removed in the postprocessing stage.

Figure 9 displays a test result carried out for an RGB-D sequence with some very jerky camera movements. (The BundleFusion codes were courteously provided by [22]. As shown in Figure 9a, the BundleFusion method successfully generated globally consistent pose estimates despite the intractable camera motions. However, we observed that frames corresponding to the fast and abrupt camera movements sometimes influence negatively on the quality of 3D reconstruction via the global optimization process as revealed in Figure 9b. On the other hand, the similarity graph-based technique focused more on finding a set of interrelated frames that would collectively create a better quality of 3D reconstruction (compare Figure 9b and Figure 9c).

### 6.5. Towards 3D World Modeling in a Mixed-Reality Environment

Despite the effort to select good pairs of frames offering stable camera tracking, the pose estimation errors from our method are inevitably accumulated over time. In general, this is true for most sophisticated state-of-the-art camera tracking models when the scan area grows beyond their capability, sometimes with jerky camera movements. Note that, given a sequence of RGB-D images, the similarity graph method uses only those frames that would result in robust camera tracking and 3D reconstruction. Thus, depending on how a scene is scanned, it often produces a collection of separate components that correspond to faithfully reconstructed, local surface regions. While additional scanning would provide extra information for connecting them automatically, another approach worth investigating is to let the user assemble the well-built components manually using the real world for guidance.

**Figure 10:**
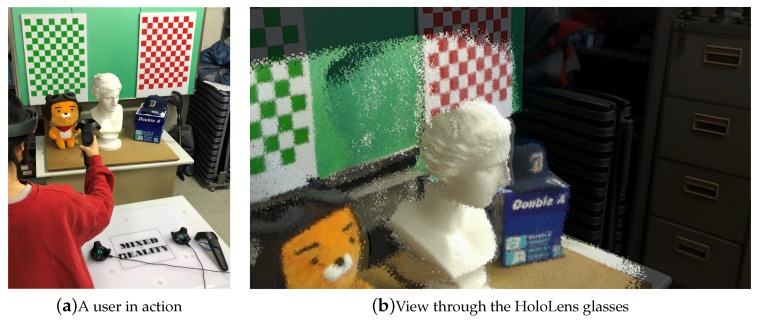
3D world modeling using the mixed-reality technology. By placing each part of the scanned geometry on the real object in a mixed-reality environment, we could effectively build 3D models for the real-world scene.

**Figure 11:**
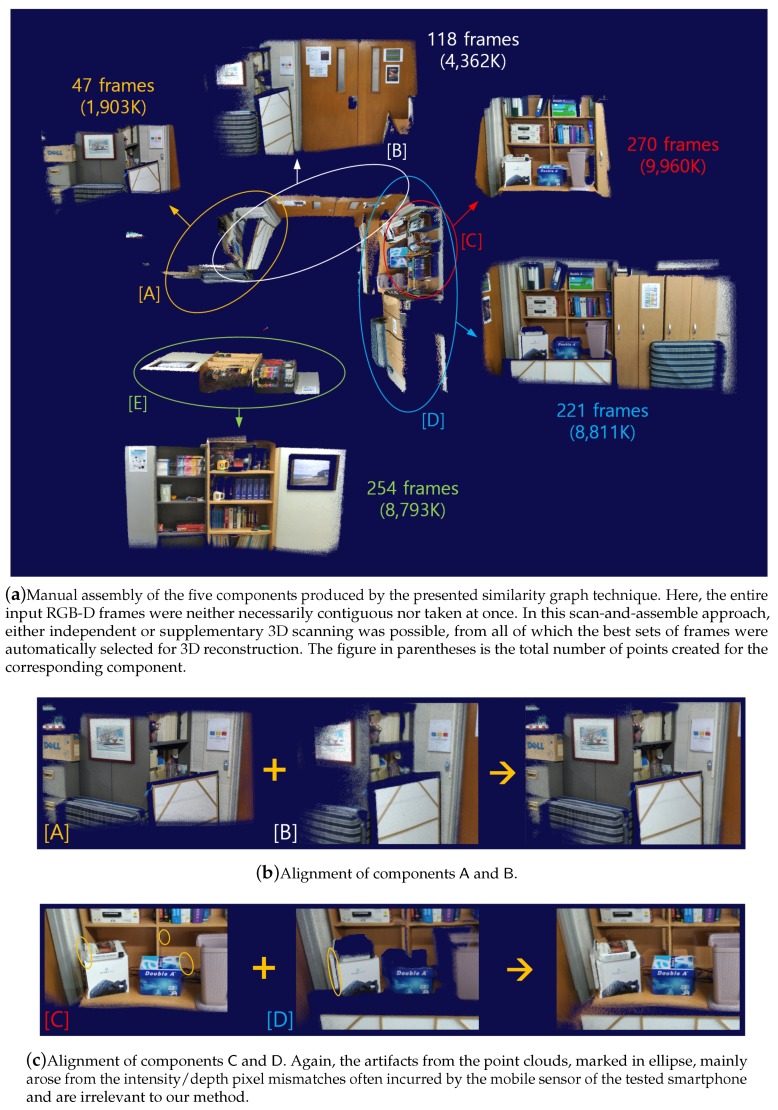
Indoor scanning of an office space using a Google Tango-enabled smartphone. In (**a**), we directly placed each of the five components from the similarity graph method on the real objects using the described experimental mixed-reality technique. As revealed in (**b**) and (**c**), the manual positioning of the parts through the HoloLens display, whose holographic image was sometimes ambiguous depending on lighting condition, achieved a nice alignment of the point clouds. Consequently, this led to a quite satisfactory 3D reconstruction result using the rather old smartphone equipped with a low-end RGB-D sensor of resolution 320×180 pixels. Note that the rigid transformation found in the global space for each component may also be used as a good initial value for further fine-tuning the relative geometric relations between the components, enhancing the 3D reconstruction quality further. An effective method for this remains to be devised.

Figure 10 shows the 3D modeling methodology that is currently being used to test our method in an experimental mixed-reality environment. Here, we set up the Microsoft HoloLens system to share physical space with the HTC Vive system so that the user wearing a HoloLens headset can freely use Vive’s controllers. Then, the user interactively selects each component of reconstructed 3D geometry and places it on the real object finely while seeing both virtual and real geometries through the holographic glasses. In this way, the user can utilize the mixed-reality technology to convert the real world into geometric models, which can in turn be used effectively to develop various mixed-reality applications (see Figure 11 for an example 3D scene modeling).

### 6.6. Towards Progressive 3D Reconstruction from a Live RGB-D Stream

**Figure 12:**
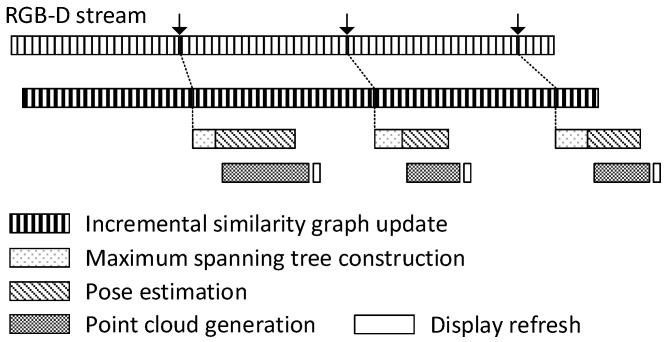
Progressive 3D reconstruction from live RGB-D streams. When a mobile device with limited computational capability is used to scan a scene, the proposed computation may be performed on PCs that are remotely connected through a communication network.

The presented method is basically an offline approach, which requires all RGB-D frames to be available for 3D reconstruction. As shown in Table 1 in Section 6.1, the method spends most of its computation time on building the similarity graph. However, the graph construction can be done incrementally in parallel with the image acquisition process so that, for every incoming frame, the similarity measure is evaluated for each of the preceding frames, adding the respective weighed edge to the graph. Figure 12 proposes a computational pipeline, in which the user would be able to check the progressively growing 3D geometry on the fly and accordingly decide which region to scan further for more effective 3D reconstruction. In this scenario, an independent CPU thread iteratively takes each incoming frame and updates the similarity graph ( Incremental similarity graph update), which can be easily accelerated on the GPU. At every given time interval (e.g., at every 30 frames), as soon as the graph update is over, another CPU thread starts finding a maximum spanning tree (Maximum spanning tree construction), and performs the frame-to-frame pose estimation indicated by the tree (Pose estimation).

The new tree usually shares many edges with the earlier tree built in the previous round. Thus, it is sufficient to carry out the registration task only for the newly found tree edges, whose computation can also be parallelized on multithreaded CPU hardware. When the camera pose for a frame has been modified or newly generated, a per-frame point set is created for 3D reconstruction from its RGB-D image using yet another CPU thread ( Point cloud generation). Once all the needed point sets are collected for display, the screen is refreshed (Display refresh).

## 7. Conclusions

In this paper, we proposed a novel approach that reconstructs 3D geometry from a stream of RGB-D images taken by a consumer-grade mobile RGB-D camera. Although our method is not confined to low-cost mobile sensors, such as the one on the tested smartphone, its low pixel resolution and limited depth precision often hindered high-quality 3D reconstruction. Therefore, the presented similarity graph technique was designed to carefully select only the frames and their registration order from the input sequence that would produce accurate pose estimation and robust 3D reconstruction.

The proposed method sometimes produced multiple separate components of 3D reconstruction in spite of the effort to automatically merge them via the component-wise camera tracking technique (Section 5.2). This was often due to the fact that there were not RGB-D frames which would help connect the disconnected components with sufficient precision using our, basically local, pose estimation technique. One solution to this would be to apply a state-of-the-art global optimization method for the pose graph constructed over the set of well-built point clouds. On the other hand, another effective solution was, as proposed in Section 6.5, to allow the user to interactively put the point cloud of each component in place with real objects for guidance in the mixed-reality environment. As the mobile RGB-D sensors and the mixed-reality devices are evolving rapidly, we believe that the 3D modeling method based on the latter approach is prospective, which will enable a smartphone user to scan his/her environments easily with the help of mixed-reality technology.

## Figures and Tables

**Table 1:** Computational costs for the similarity graph-based pose estimation (unit: sec). For each stream made of $n_{fr}$ RGB-D images of 320×180 pixels, the four columns titled by “sim-gr”, “mst”, “paths”, and “pose-est” respectively represent the total times consumed in the four major steps of our method: the construction of similarity graph, the construction of maximum spanning tree, the extraction of frame pair sequences via the tree traversal, and the actual frame-to-frame camera tracking. In the parentheses in the fifth column, the average per-frame pose estimation times are given. Finally, the last column “amortized” indicates the amortized per-frame tracking costs reflecting all computations.

$n_{\mathrm{fr}}$	Sim-Gr	Mst	Paths	Pose-Est	Amortized
200	0.77	0.0068	0.0022	5.15 (0.0258)	0.0297
400	2.86	0.0332	0.0044	10.62 (0.0266)	0.0338
800	10.83	0.1879	0.0128	22.59 (0.0282)	0.0420
1600	40.52	1.0450	0.0520	46.50 (0.0291)	0.0551

## References

[1] Zollhöfer M., Stotko P., Görlitz A., Theobalt C., Nießner M., Klein R., Kolb A. (2018). State of the art on 3D reconstruction with RGB-D cameras. Comput. Graph. Forum.

[2] Triggs B., McLauchlan P.F., Hartley R.I., Fitzgibbon A.W. Bundle adjustment—A modern synthesis. Proceedings of the International Workshop on Vision Algorithms: Theory and Practice.

[3] Kümmerle R., Grisetti G., Strasdat H., Konolige K., Burgard W. G^2^o: A general framework for graph optimization. Proceedings of the 2011 IEEE International Conference on Robotics and Automation.

[4] Lee G., Fraundorfer F., Pollefeys M. Robust pose-graph loop-closures with expectation-maximization. Proceedings of the 2013 IEEE/RSJ International Conference on Intelligent Robots and Systems.

[5] Newcombe R.A., Izadi S., Hilliges O., Molyneaux D., Kim D., Davison A.J., Kohi P., Shotton J., Hodges S., Fitzgibbon A. KinectFusion: Real-time dense surface mapping and tracking. Proceedings of the 2011 10th IEEE International Symposium on Mixed and Augmented Reality.

[6] Roth H., Vona M. Moving volume KinectFusion. Proceedings of the British Machine Vision Conference.

[7] Whelan T., McDonald J., Kaess M., Fallon M., Johannsson H., Leonard J. Kintinuous: Spatially extended KinectFusion. Proceedings of the RSS Workshop on RGB-D: Advanced Reasoning with Depth Cameras.

[8] Chen J., Bautembach D., Izadi S. (2013). Scalable real-time volumetric surface reconstruction. ACM Trans. Graph..

[9] Nießner M., Zollhöfer M., Izadi S., Stamminger M. (2013). Real-time 3D reconstruction at scale using voxel hashing. ACM Trans. Graph..

[10] Steinbrücker F., Sturm J., Cremers D. Real-time visual odometry from dense RGB-D images. Proceedings of the 2011 IEEE International Conference on Computer Vision Workshops (ICCV Workshops).

[11] Audras C., Comport A., Meilland M., Rives P. Real-time dense appearance-based SLAM for RGB-D sensors. Proceedings of the Australasian Conference on Robotics and Automation (ACRA).

[12] Tykkälä T., Audras C., Comport A. Direct iterative closest point for real-time visual odometry. Proceedings of the 2011 IEEE International Conference on Computer Vision Workshops (ICCV Workshops).

[13] Glocker B., Shotton J., Criminisi A., Izadi S. (2015). Real-time RGB-D camera relocalization via randomized ferns for keyframe encoding. IEEE Trans. Vis. Comput. Graph.

[14] Endres F., Hess J., Engelhard N., Sturm J., Cremers D., Burgard W. An evalution of the RGB-D SLAM system. Proceedings of the 2012 IEEE International Conference Robotics Automation (ICRA).

[15] Henry P., Krainin M., Herbst E., Ren X., Fox D. (2012). RGB-D mapping: Using Kinect-style depth cameras for dense 3D modeling of indoor environments. Int. J. Robot. Res..

[16] Stückler J., Behnke S. Integrating depth and color cues for dense multi-resolution scene mapping using RGB-D cameras. Proceedings of the 2012 IEEE International Conference on Multisensor Fusion and Integration for Intelligent Systems (MFI).

[17] Kerl C., Sturm J., Cremers D. Dense visual SLAM for RGB-D cameras. Proceedings of the 2013 IEEE/RSJ International Conference on Intelligent Robots and Systems (IROS).

[18] Maier R., Sturm J., Cremers D. Submap-based bundle adjustment for 3D reconstruction from RGB-D data. Proceedings of the German Conference on Pattern Recognition.

[19] Stückler J., Behnke S. (2014). Multi-resolution surfel maps for efficient dense 3D modeling and tracking. J. Vis. Commun. Image Represent..

[20] Choi S., Zhou Q., Koltun V. Robust reconstruction of indoor scenes. Proceedings of the 2015 IEEE Conference on Computer Vision and Pattern Recognition (CVPR).

[21] Kähler O., Prisacariu V., Murray D. Real-time large-scale dense 3D reconstruction with loop closure. Proceedings of the 2016 European Conference on Computer Vision.

[22] Dai A., Nießner M., Zollöfer M., Izadi S., Theobalt C. (2017). BundleFusion: Real-time globally consistent 3D reconstruction using on-the-fly surface re-integration. ACM Trans. Graph..

[23] Cao Y., Kobbelt L., Hu S. (2018). Real-time high-accuracy three-dimensional reconstruction with consumer RGB-D cameras. ACM Trans. Graph..

[24] Keller M., Lefloch D., Lambers M., Izadi S., Weyrich T., Kolb A. (2013). Real-time 3D reconstruction in dynamic scenes using point-based fusion. Proceedings of the 2013 International Conference on 3D Vision.

[25] Ruhnke M., Kümmerle R., Grisetti G., Burgard W. Highly accurate 3D surface models by sparse surface adjustment. Proceedings of the 2012 IEEE International Conference on Robotics and Automation.

[26] Whelan T., Kaess M., Leonard J.J., McDonald J. Deformation-based loop closure for large scale dense RGB-D SLAM. Proceedings of the 2013 IEEE/RSJ International Conference on Intelligent Robot Systems.

[27] Whelan T., Salas-Moreno R., Glocker B., Davison J., Leutenegger S. (2016). ElasticFusion: Real-time dense SLAM and light source estimation. Int. J. Robot. Res..

[28] Mur-Artal R., Tardós J.D. (2017). ORB-SLAM2: An open-source SLAM system for monocular, stereo, and RGB-D cameras. IEEE Trans. Robot..

[29] Yousif K., Taguchi Y., Ramalingam S. MonoRGBD-SLAM: Simultaneous localization and mapping using both monocular and RGBD cameras. Proceedings of the 2017 IEEE International Conference on Robotics and Automation (ICRA).

[30] Endres F., Hess J., Sturm J., Cremers D., Burgard W. (2014). 3-D mapping with an RGB-D camera. IEEE Trans. Robot..

[31] An J., Lee J., Jeong J., Ihm I. Tracking an RGB-D camera on mobile devices using an improved frame-to-frame pose estimation method. Proceedings of the IEEE Winter Conference on Applications of Computer Vision 2018 (WACV 2018).

[32] Solomon J. (2015). Numerical Algorithms: Methods for Computer Vision, Machine Learning, and Graphics.

[33] Sedgewick R. (2002). Algorithms in C: Part 5 Graph Algorithms.

[34] Handa A., Whelan T., McDonald J., Davison A. A benchmark for RGB-D visual odometry, 3D reconstruction and SLAM. Proceedings of the IEEE International Conference on Robotics and Automation.

[35] Sturm J., Engelhard N., Endres F., Burgard W., Cremers D. A benchmark for the evaluation of RGB-D SLAM systems. Proceedings of the International Conference on Intelligent Robot Systems.

